# Early Prediction of Intensive Care Unit–Acquired Weakness Using Easily Available Parameters: A Prospective Observational Study

**DOI:** 10.1371/journal.pone.0111259

**Published:** 2014-10-27

**Authors:** Luuk Wieske, Esther Witteveen, Camiel Verhamme, Daniela S. Dettling-Ihnenfeldt, Marike van der Schaaf, Marcus J. Schultz, Ivo N. van Schaik, Janneke Horn

**Affiliations:** 1 Department of Intensive Care Medicine, Academic Medical Center, Amsterdam, the Netherlands; 2 Department of Neurology, Academic Medical Center, Amsterdam, the Netherlands; 3 Laboratory of Experimental Anesthesiology and Intensive Care (L•E•I•C•A), Academic Medical Center, Amsterdam, the Netherlands; 4 Department of Rehabilitation, Academic Medical Center, Amsterdam, the Netherlands; D’or Institute of Research and Education, Brazil

## Abstract

**Introduction:**

An early diagnosis of Intensive Care Unit–acquired weakness (ICU–AW) using muscle strength assessment is not possible in most critically ill patients. We hypothesized that development of ICU–AW can be predicted reliably two days after ICU admission, using patient characteristics, early available clinical parameters, laboratory results and use of medication as parameters.

**Methods:**

Newly admitted ICU patients mechanically ventilated ≥2 days were included in this prospective observational cohort study. Manual muscle strength was measured according to the Medical Research Council (MRC) scale, when patients were awake and attentive. ICU–AW was defined as an average MRC score <4. A prediction model was developed by selecting predictors from an a–priori defined set of candidate predictors, based on known risk factors. Discriminative performance of the prediction model was evaluated, validated internally and compared to the APACHE IV and SOFA score.

**Results:**

Of 212 included patients, 103 developed ICU–AW. Highest lactate levels, treatment with any aminoglycoside in the first two days after admission and age were selected as predictors. The area under the receiver operating characteristic curve of the prediction model was 0.71 after internal validation. The new prediction model improved discrimination compared to the APACHE IV and the SOFA score.

**Conclusion:**

The new early prediction model for ICU–AW using a set of 3 easily available parameters has fair discriminative performance. This model needs external validation.

## Introduction

Intensive Care Unit–acquired weakness (ICU–AW) is a frequent and debilitating neuromuscular complication of critical illness. [1], [2] Development of ICU-AW is associated with increased mortality and short- and long term morbidity. [3]–[5] Currently, no specific treatments for ICU-AW exist. For future treatments to be successful, timing may be of importance. The first signs of ICU-AW can be found starting from day 2 after admission when decreased excitability of muscle and nerve can be observed. [6], [7] Initiation of treatment at this moment may be more effective because the observed abnormalities may still be reversible. [8], [9] Such early treatment would require an early diagnosis of ICU-AW. At present, the diagnosis of ICU–AW is based on clinical examination using manual muscle strength assessment. [1] In most critically ill patients, manual muscle strength assessment is not possible early in the disease course due to impaired consciousness or attentiveness. [10] A solution to this diagnostic delay may be to quantify the risk that a patient will develop ICU–AW using a prediction model early after ICU admission.

ICU–AW is associated with several risk factors, including sepsis, the presence of multiple organ dysfunction syndrome (MODS) and severity of illness. [11] Prediction of ICU–AW on the basis of these risk factors is scarcely studied. A combination of the Acute Physiology and Chronic Health Evaluation (APACHE) score and presence of the Systemic Inflammatory Response Syndrome (SIRS) could identify patients at high risk for development of ICU-AW, although the predictive performance was not reported. [12] A cumulative Sequential Organ Failure Assessment (SOFA) score of the first week of ICU admission also has predictive value but this approach does not allow early prediction [13].

We hypothesized that early prediction of ICU–AW is possible and reliable. To investigate this, we built a prediction model based on previously identified risk factors for ICU–AW. The predictive performance of the model was compared to those of the APACHE IV scores and the SOFA score.

## Methods

### Design and ethical approval

We performed a prospective observational cohort study using the STARD guidelines. [14] The institutional review board of the Academic Medical Center, Amsterdam, The Netherlands, decided (decision notice W13_080#13.17.0100) that data for this study could be collected and analyzed without written informed consent of the patient and specifically approved the use of that data for this study because no additional procedures were performed and therefore this study did not fulfill the criteria for medical research stated in the Dutch ‘Law on medical research’.

### Study setting

The study was performed in a 30 beds tertiary mixed medical-surgical ICU of the Academic Medical Center in the Netherlands. In this ICU, several standards of care are applied including glucose control between 90 mg/dl and 144 mg/dl. Sedation is stopped as soon possible. Norepinephrine is the first line vasopressor drug and corticosteroids (100 mg of hydrocortisone intravenously 3 times daily) are given in refractory septic shock. All patients receive early rehabilitation.

### In– and exclusion criteria

Consecutive, newly admitted ICU patients, mechanically ventilated for ≥2 days, were included. We excluded patients who had a neuromuscular disorder (e.g. Guillain-Barré syndrome), stroke, out-of-hospital cardiac arrest or spinal injury as reason for ICU admission. In addition, we excluded patients with a poor pre-hospital functional status (modified Rankin scale ≥4 [15]) and patients with pre-existing spinal injury.

### Strength assessment (reference standard)

Physical therapists, blinded for all other parameters, assessed muscle strength when patients were alert (Richmond Agitation and Sedation Scale between −1 and 1 [16]) and attentive (able to follow verbal commands using arms or eye-lids). When patients are alert and attentive, muscle strength can be reliably assessed using the MRC score. [17] MRC scores were assessed bilaterally in 6 pre-specified muscle groups: wrist dorsiflexors, elbow flexors, shoulder abductors, hip flexors, knee extensors and ankle dorsiflexors. MRC scores of muscle groups were summated and divided by the number of muscle groups that could be tested to obtain an average MRC score. When a muscle group could not be assessed, no value was imputed. ICU–AW was diagnosed when weakness had developed after ICU admission, was symmetric and the average MRC score was <4 [1].

### Candidate predictors

Candidate predictors were based on risk factors for ICU-AW identified through a literature search (see material S1). We extracted risk factors that were easily available in the first two days after ICU admission and had a univariate association, in at least one study. To improve suitability for prediction, some of the extracted risk factors were redefined into candidate predictors with more clear definitions. Candidate predictors regarding medical history and the presence of suspected sepsis were scored during ICU admission; all others were obtained from the electronic patient record after ICU discharge. Candidate predictors were collected blinded for the reference standard.

### Additional data collected

The following additional clinical characteristics were collected: the Acute Physiology and Chronic Health Evaluation IV (APACHE IV) score and the maximal Sequential Organ Failure Assessment (SOFA) score during the first two days after ICU admission. Also, data on the number of days with mechanical ventilation, length of stay in the ICU and ICU mortality were collected.

### Sample size

We assumed that ICU–AW would occur in 50% of patients [11] and that 5 patients with ICU–AW needed to be included per candidate predictor. We defined a set of 20 candidate predictors, so 200 patients were needed.

### Statistical analysis

Candidate predictors with right-tailed distributions were logarithmically transformed. Predictors for the model were selected from candidate predictors using two steps. First, using a bootstrapped backward selection process, candidate predictors included in ≥50% of the bootstrap samples (N:1000; p<0.5 for inclusion) were selected. [18], [19] Next, the selected candidate predictors were consecutively entered in a logistic regression model and only those candidate predictors that led to a discriminatory increase in model fit were retained. Candidate predictors were entered in descending order of inclusion frequency in bootstrap samples. For every addition, the change in Akaike Information Criterion (AIC) between models was compared. [20] An AIC change >–2 between additions was interpreted as non-discriminatory and the candidate predictors included before that addition were selected as predictors.

Next, we constructed a model with these predictors. Discriminative performance was analyzed using the area under the Receiver Operating Characteristic (AUC–ROC) curve and internally validated using bootstrapping (N:1000). We defined AUC-ROC values between 0.90–1 as excellent, 0.80–0.90 as good, 0.70–0.80 as fair, 0.60–0.70 as poor and <0.60 as failed. Odds ratios were adjusted using the calibration slope after internal validation. [18] Calibration was assessed graphically and using goodness of fit (Hosmer–Lemeshow test).

Performance of the new prediction model was compared to the APACHE IV score and maximal SOFA score in the first two ICU days using continuous net reclassification improvement (cNRI), which is a measure of discrimination resembling the AUC-ROC but more sensitive to change [21].

Finally, we performed two sensitivity analyses; we investigated discrimination of the prediction model for more severe ICU-AW (ICU-AW defined using a lower cut-off, i.e. an average MRC<3). Second, we investigated the influence of missing data by repeating predictor selection and model discrimination analyses on data sets in which missing data was imputed using multivariate imputation by chained equations (10 iterations of 10 imputations). [22] For the imputation model, all 20 candidate predictors as well as the presence of ICU-AW were used. Imputed values were checked for validity.

Mean values are presented with standard deviation (±SD), median values with interquartile range (IQR) and proportions with percentages and total numbers. Differences between proportions were assessed using chi-square test. Differences between normally distributed variables were assessed using Welch’s t-test; differences between non-normally distributed continuous variables were assessed using Wilcoxon rank-sum test. Analyses were done using R (version: 2.15.2).

## Results

### Patients

**Figure 1:**
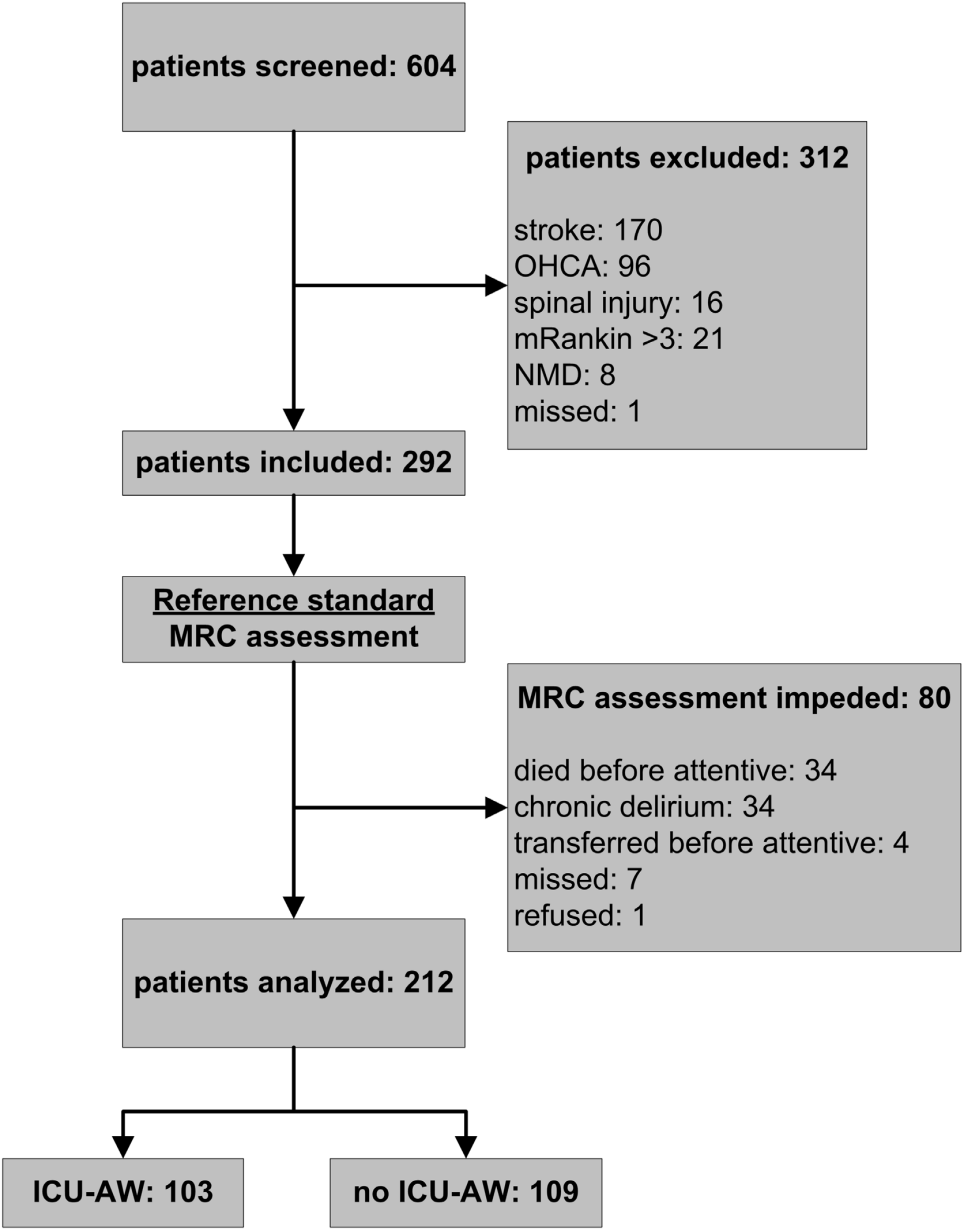
Study flowchart. ICU-AW: Intensive Care Unit – acquired weakness; OHCA: out-of hospital cardiac arrest; mRankin: modified Rankin score; NMD: neuromuscular disorder; MRC: muscle strength as assessed with Medical Research Council scale.

Figure 1 displays the flow chart. Patients were screened from January 2011 until December 2012. Muscle strength could be assessed in 212 patients. Of those patients, 103 patients (49%) were diagnosed with ICU–AW. Table 1 shows patient and admission characteristics.

**Table 1:** Patient and admission characteristics.

	ICU-AW(N:103)	no ICU-AW(N:109)	p-value
age, mean ± SD	63±15	59±16	0.08
females, n (%)	52 (50)	40 (37)	0.06
reason for admission: planned surgical, n (%)	18 (17)	26 (24)	
reason for admission: emergency surgical, n (%)	28 (27)	21 (19)	0.29
reason for admission: medical, n (%)	57 (55)	62 (57)	
APACHE IV score, mean ± SD (3 missing)	89±25	74±28	<0.01
maximal SOFA score in first two days, mean ± SD	11±3	9±3	<0.01
average MRC score, median (IQR)	2.5 (1.3 to 3.2)	4.8 (4 to 5)	n.a.
day of MRC assessment after ICU admission,median (IQR)	9 (6–16)	7 (5–9)	<0.01
days with MV, median days (IQR)	13 (6 to 22)	6 (4 to 8)	<0.01
LOS ICU, median days (IQR)	16 (9 to 28)	8 (6 to 11)	<0.01
ICU mortality, n (%)	35 (34)	10 (9)	<0.01

ICU-AW: Intensive Care Unit – acquired weakness; LOS ICU: length of stay in the intensive care unit; APACHE IV: Acute Physiology and Chronic Health Evaluation IV; SOFA: Sequential Organ Failure Assessment; MV: mechanical ventilation; MRC: Medical Research Council; n.a.: not applicable.

### Candidate predictors

The literature search identified two systematic reviews on risk factors for ICU-AW. [11], [23] Three additional recently published cohort studies were found. [24]–[26] We extracted 17 risk factors (see material S1) and redefined some of the risk factors so that they were suitable as candidate predictors (see figure S1). Three extra candidate predictors that have never been investigated but are likely to be of importance in ICU-AW were added, i.e. presence of pre-existing polyneuropathy, presence of risk factors for polyneuropathy and systemic corticosteroid use prior to ICU admission. All 20 candidate predictors are displayed in table 2; table S1 displays descriptions and definitions.

**Table 2:** Candidate predictors for development of prediction model for early prediction of Intensive Care Unit – acquired weakness.

candidate predictors*	distribution		p-value	selection percentage inbootstrap samples
	ICU-AW(N:103)	no ICU-AW(N:109)		
**patient characteristics**				
females, n (%)	52 (50)	40 (37)	0.06	37.1
age, mean ± SD	63±15	59±16	0.08	57.6
risk factor for a polyneuropathyin medical history, n (%)	35 (34)	40 (37)	0.79	13.4
pre-existing polyneuropathy priorto ICU admission, n (%)	3 (3)	1 (1)	0.57	n.a.
systemic corticosteroid use priorto ICU admission, n (%)	7 (7)	9 (8)	0.89	10.7
**clinical parameters**				
suspected sepsis, n (%)	78 (76)	70 (64)	0.09	14.7
unplanned admission, n (%)	85 (83)	83 (76)	0.33	10.9
presence of shock, n (%)	75 (73)	67 (61)	0.11	24.6
RASS score, median (IQR)	–3 (–5 to −1)	–2 (–3 to 0)	<0.01	48.2
**laboratory parameters**				
average urine production,median ml/h (IQR)	70 (20 to 122)	102(64 to 134)	<0.01	14.4
highest glucose, mean mg/dl ± SD	243.8±78.5	220.5±67.3	0.02	38.5
lowest glucose, mean mg/dl ± SD	85.8±22.3	89.6±25.8	0.25	22.2
lowest pH, mean ± SD	7.21±0.1	7.25±0.1	0.02	17.3
lowest P/F ratio, median (IQR)	186 (127 to 245)	178 (134 to 246)	0.98	27.9
lowest platelet count,median×10^9^/L (IQR)	103 (45 to 151)	127 (85 to 197)	0.01	21.0
highest lactate, median mmol/L(IQR; 17 missing)	4.5 (3.0 to 7.0)	2.8 (1.7 to 4.8)	<0.01	89.5‡
lowest ionized Ca^2+^, meanmmol/L ± SD	0.97±0.11	0.98±0.13	0.53	51.6
**medication**				
treatment with any corticosteroid,n (%)	81 (79)	63 (58)	<0.01	33.9
repeated treatment with anyneuromuscular blocker§, n (%)	17 (17)	18 (17)	1.00	20.3
treatment with anyaminoglycoside, n (%)	51 (50)	30 (28)	<0.01	80.4

*all clinical, laboratory and medication parameters were scored using information from the first two ICU days, except for the RASS score which was scored around two days after ICU admission;

‡ logarithmically transformed;

§ more than one administration of any neuromuscular blocker.

ICU-AW: Intensive Care Unit – acquired weakness; RASS: Richmond Agitation and Sedation Scale; n.a.: not applicable.

Table displaying distributions and differences between patients with and without Intensive Care Unit – acquired weakness for the candidate predictors. In the final column selection percentages of the candidate predictors in bootstrap samples based on backward selection are presented.

### Predictor selection

Table 2 gives an overview of the distributions of the different candidate predictors for patients who did or did not develop ICU–AW. Because of the low number of patients with a pre–existing polyneuropathy, this candidate predictor was excluded from predictor selection. After backward selection, highest lactate, treatment with any aminoglycoside, age and lowest ionized Ca^2+^ were included in ≥50% of bootstrap samples (table 2). After consecutive addition of these candidate predictors into a logistic regression model, addition of lowest ionized Ca^2+^ did not result in discriminatory change in AIC (table 3). Therefore, highest lactate, treatment with any aminoglycoside and age were selected as predictors.

**Table 3:** Construction of prediction model.

candidate predictors	selection percentagein bootstrap samples	change inAIC	multivariate OR(95%-CI)	adjustedmultivariate OR†
highest lactate‡(17 missing)	89.5	n.a.	2.18 (1.39 to 3.43)	2.08
treatment with anyaminoglycoside	80.4	–5.8	2.75 (1.44 to 5.26)	2.59
age	57.6	–2.8	1.02 (1.00 to 1.04)	1.02
lowest ionized Ca^2+^	51.6	–1.6	not included	n.a.

‡ logarithmically transformed.

† adjusted for overfitting using a shrinkage factor (i.e. calibration slope) of 0.94 obtained after internal validation.

n.a.: not applicable; AIC: Akaike Information Criterion; OR: odds ratio; CI: confidence interval.

Candidate predictors that were included in ≥50% of bootstrap samples (table 2) were entered consecutively into a logistic regression model starting with the most selected candidate predictor. For every subsequent step, the change in Akaike Information Criterion (AIC) was compared and candidate predictors were only included in the prediction model if addition resulted in a change in AIC<–2. In the final columns unadjusted and adjusted multivariate odds ratio’s for predictors included in the prediction model are presented.

### Prediction model

**Figure 2:**
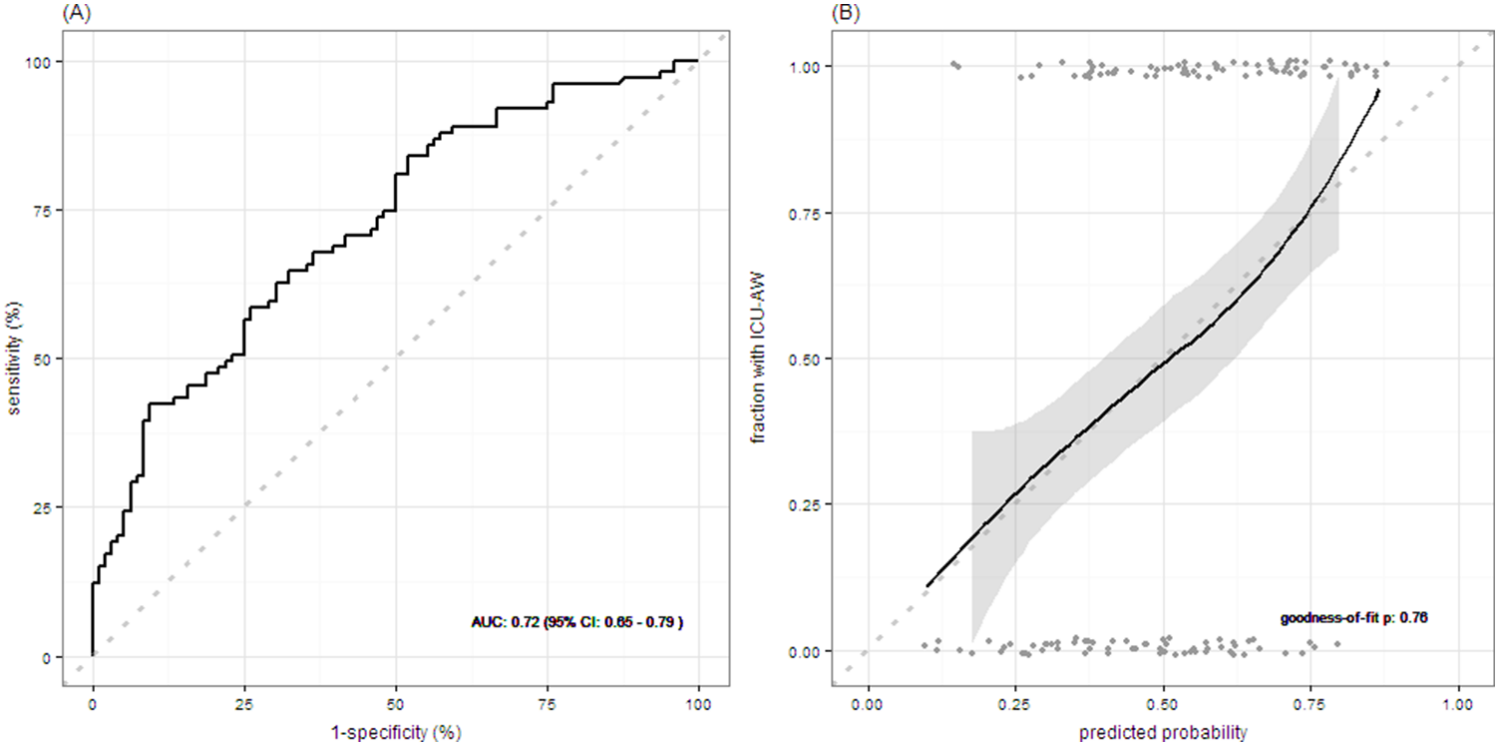
Model performance for early prediction of Intensive Care Unit – acquired weakness. Panel A shows the receiver operating characteristic (ROC) curve assessing discrimination of the prediction model. Panel B shows model calibration assessed with a fitted curve based on loess regression with 95% confidence interval (perfect model calibration is illustrated by the dotted line). Goodness-of-fit assessed with the Hosmer–Lemeshow test is shown. Grey points represent predicted probabilities for individual patients. AUC: area under the receiver operating characteristic curve; CI: confidence interval; ICU-AW: Intensive Care Unit – acquired weakness.

Table 3 shows the multivariate odds ratios for the 3 predictors, both unadjusted and adjusted for overfitting. The AUC–ROC of the prediction model was 0.72 (95%-CI: 0.65–0.79; panel A figure 2) and decreased to 0.71 after interval validation. The model showed good calibration (panel B of figure 2) without evidence for lack of fit. A spreadsheet calculator based on the prediction model is provided as material S2.

### Comparison with APACHE IV and SOFA scores

The AUC-ROC of the maximal SOFA score in the first two ICU days for prediction of ICU-AW was 0.64 (95%-CI: 0.57–0.72); the AUC-ROC of the APACHE IV score was 0.66 (95%-CI: 0.58–0.73). Discrimination improved when using the new prediction model, both when compared to the maximal SOFA score in the first two ICU days and APACHE IV score (cNRI: 34% (95%-CI: 6 to 62) and 48% (95%-CI: 20 to 75), respectively).

### Sensitivity analyses

For prediction of more severe ICU–AW (severe ICU-AW defined as an average MRC score <3; 64 of 212 patients met this definition), discriminative performance of the prediction model was not different (AUC-ROC: 0.72).

Highest lactate levels were missing in 17 patients; no other parameters had missing values. When repeating the backward selection process on data sets with missing lactate levels imputed, the same candidate predictors had a selection frequency of ≥50% and no additional candidate predictors were identified. Furthermore, based on change in AIC, addition of lowest ionized Ca^2+^ was non-discriminatory in all the imputation models. The discriminative performance of the prediction model was not different (averaged AUC–ROC after internal validation: 0.71) in the imputed data sets.

## Discussion

After the first two days of stay in the ICU, development of ICU-AW can be predicted using highest lactate levels, treatment with any aminoglycoside and age as predictors. Discriminative performance of the prediction model was fair.

### Comparison with previous studies

This is the first prediction model that has been developed specifically for early prediction of ICU-AW. When compared to previously identified predictors for ICU-AW, i.e. the APACHE and SOFA scores, the new prediction model had better discriminative performance [12], [13].

Other, more technically demanding, methods for early prediction of ICU-AW have also been investigated. Weber-Carstens et al studied early electrophysiological testing and found a sensitivity of 83% and specificity of 89% for direct muscle stimulation. [27] This is indicative of a better discriminative performance than our prediction model, but electrophysiological studies in general, and direct muscle stimulation in particular, are technically demanding and are not widely available in ICUs. [1] Diagnostic potential of other methods for an early diagnosis of ICU-AW, like ultrasound or biological markers, has been scarcely studied [1], [28], [29].

### Biological plausibility of the prediction model

Several hypotheses have been proposed that provide biological plausibility for the predictors that have been included in our model. Bolton proposed that tissue hypoxia caused by impaired microcirculation, for which lactate levels are a marker, is involved in pathogenesis of ICU-AW. [30], [31] Aminoglycosides may be involved in ICU-AW because they can impair neuromuscular transmission and because of their neurotoxicity. [30], [32] With aging, there is an accumulating burden of (neuromuscular) co-morbidities, a physiological loss of skeletal muscle mass and a decrease in mobility; all of which could lead to an increased susceptibility to develop ICU-AW. [33] All of these hypotheses remain speculative since none have been investigated properly.

We would like to emphasize that a good discriminatory performance of a predictor does not mean that this predictor also plays a (important) role in the pathogenesis of ICU-AW. Prediction model analyses do not require multivariate analyses to assess an independent association between a variable and the outcome, from which a causal relation may be inferred. [34] For prediction, only the discriminatory performance is important. Sepsis for example was not selected although it is a well-known risk factor for ICU-AW because it was not discriminatory in our population as it was highly prevalent in both patients with and without ICU-AW.

### Strengths and limitations

Strengths of this study are the inclusion of a diagnostically relevant population and the use of easily available predictors. Our study also has limitations. First, the number of candidate predictors was larger than the general rule of thumb of 1 candidate predictor for 10 events. [34] Although our candidate predictors were based on previously identified risk factors, this does not necessarily mean that these parameters are also good predictors. We expected that not all candidate predictors would have predictive value and therefore decided to include more candidate predictors than is recommended. To reduce the subsequent risk of overfitting, we performed a bootstrapped backward selection process, followed by an additional selection step based on model fit. The prediction model we developed and evaluated was adequately powered, with 1 predictor per 33 events and a modest degree of overfitting evident after internal validation. Second, we chose a liberal p-value for inclusion in the prediction model to prevent erroneous elimination of “true” predictors. [18] This may however increase the risk of including “noise” predictors. Finally, we chose not to include composite candidate predictors, like existing severity of illness or organ failures scores (for example APACHE or SOFA). These existing scores contain several variables that are not associated with ICU-AW or include variables that we already included. Therefore, adding these scores as a whole would have led to the inclusion of variables twice or variables with no discriminatory value. A large and inefficient dataset would have been needed to feed the model. Our goal was to only add simple candidate predictors with a unique discriminatory value in order to keep the data set necessary as small and efficient as possible.

### Framework for future studies

The ability to predict ICU-AW early after ICU admission and circumvent this limitation of muscle strength assessment as a diagnostic method can be an important step in critical care and research. Our study indicates that this prediction model, using easily available predictors, may be an option to achieve this. However, we did not investigate external validity, which is mandatory to ascertain the true discriminative performance of a prediction model. [35] It will be important to externally validate this prediction model in a multicenter setting to maximize generalizability. The discriminative performance after external validation, possibly recalibrated and updated with new predictors in future, will determine the true value of this model and whether or not it can be used in the clinic.

If the model is found to be reliable enough for clinical application it may be used to improve prognostication and to guide patient management. Also, prediction may be used to start therapies early, before structural damage to nerves and muscles has occurred, which is thought to possibly increase treatment effects. [10] Currently, no high quality evidence is available supporting an intervention for ICU-AW but some prospects exist. Early mobilization, starting when patients are still sedated, may be effective for preventing ICU-AW. [36] Early administration of intravenous immunoglobulins did not prevent ICU-AW. [37] Other pharmacological options, like melatonin, oxytocin, levetiracetam, indomethacin and leupeptin, have only been investigated in animals models. [38]–[40] Clinical research is needed to confirm these observations and to find new therapeutic options.

## Conclusion

Early prediction of ICU–AW is possible using a set of 3 easily available predictors. Discriminative performance of the prediction model seems fair but needs external validation.

## Supporting Information

Figure S1 **Redefining of relevant risk factors into candidate predictors.**
(PDF)

Table S1 **Descriptions and definitions for candidate predictors.**
(PDF)

Material S1 **Supporting text concerning selection of candidate predictors.**
(PDF)

Material S2 **Spreadsheet calculator.**
(XLS)

Material S3 **Data set.**
(CSV)
