# Supplementary material for: Early Prediction of Intensive Care Unit–Acquired Weakness Using Easily Available Parameters: A Prospective Observational Study
Source: PLoS One. 2014 Oct 27;9(10):e111259. doi: 10.1371/journal.pone.0111259 (PMC4210178; doi:10.1371/journal.pone.0111259)
Supplement: Figure S1 — Redefining of relevant risk factors into candidate predictors. (PDF) [file pone.0111259.s001.pdf]

Figure S1: Redefining of relevant risk factors into candidate predictors

Figure displaying the translation of identified risk factors into clinically usable candidate predictors. The overlapping arrows indicate that one risk factor may contribute to one or more candidate predictors and that candidate predictors may be translated from one or more identified risk factors.

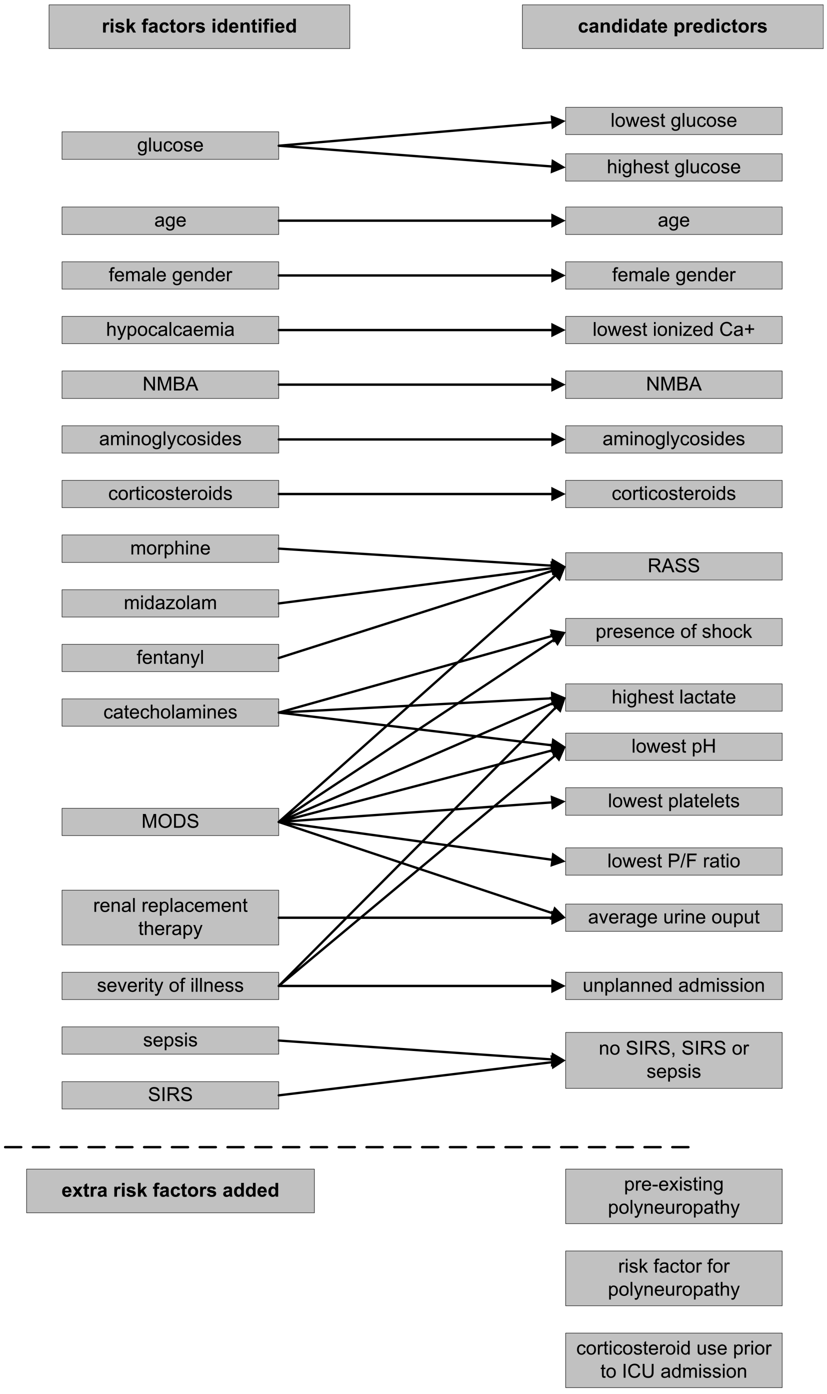

SIRS: systemic inflammatory response syndrome; RASS: Richmond Agitation and Sedation Scale; MODS: multiple organ dysfunction syndrome, NMBA: neuromuscular blocking agents.
