# Supplementary material for: Early Prediction of Intensive Care Unit–Acquired Weakness Using Easily Available Parameters: A Prospective Observational Study
Source: PLoS One. 2014 Oct 27;9(10):e111259. doi: 10.1371/journal.pone.0111259 (PMC4210178; doi:10.1371/journal.pone.0111259)
Supplement: Table S1 — Descriptions and definitions for candidate predictors. (PDF) [file pone.0111259.s002.pdf]

Table S1: Descriptions and definitions for candidate predictors

candidate predictor	class	description and definition
female gender	yes/no	
age	continuous (years)	
risk factor for a polyneuropathy in medical history	yes/no	history of diabetes, alcohol abuse, previous treatment with chemotherapy, kidney transplant or dialysis
pre-existing polyneuropathy prior to ICU admission	yes/no	any pre-existing polyneuropathy
systemic corticosteroid use prior to ICU admission	yes/no	chronic (>1 month) systemic corticosteroid use
suspected sepsis	yes/no	2 or more SIRS criteria present and a suspected infection[1] in the first two ICU days*
unplanned admission	yes/no	
presence of shock	yes/no	SOFA cardiovascular score of 4 in the first two ICU days
RASS score	ordinal scale (-5 to 4)	assessment at two days after ICU admission
average urine production	continuous (ml/h)	in the first two ICU days
highest glucose	continuous (mg/dl)	in the first two ICU days
lowest glucose	continuous (mg/dl)	in the first two ICU days
lowest pH	continuous	in the first two ICU days
lowest P/F ratio	continuous	in the first two ICU days
lowest platelet count	continuous ($x10^9/L$)	in the first two ICU days
highest lactate	continuous (mmol/L)	in the first two ICU days
lowest ionized Ca^{2+}	continuous (mmol/L)	in the first two ICU days
treatment with any corticosteroid	yes/no	in the first two ICU days
repeated treatment with any neuromuscular blocker	yes/no	more than one administration of any neuromuscular blocker in the first two ICU days
treatment with any aminoglycoside	yes/no	in the first two ICU days

*originally coded as no SIRS, SIRS or suspected sepsis but because of low number of patients no SIRS (N:4) recoded into suspected sepsis yes/no

SIRS: systemic inflammatory response syndrome; SOFA: Sequential Organ Failure Assessment; RASS: Richmond Agitation and Sedation Scale; ICU: Intensive Care Unit.

Literature for Table S1

1. American College of Chest Physicians/Society of Critical Care Medicine Consensus Conference: definitions for sepsis and organ failure and guidelines for the use of innovative therapies in sepsis. (1992). Crit Care Med 20: 864–874.
