# Supplementary material for: Early Prediction of Intensive Care Unit–Acquired Weakness Using Easily Available Parameters: A Prospective Observational Study
Source: PLoS One. 2014 Oct 27;9(10):e111259. doi: 10.1371/journal.pone.0111259 (PMC4210178; doi:10.1371/journal.pone.0111259)
Supplement: Material S1 — Supporting text concerning selection of candidate predictors. (PDF) [file pone.0111259.s003.pdf]

Supporting material S1:

Search strategy for relevant risk factors

To identify relevant risk factors, we searched for systematic reviews on risk factors for ICU-AW using the following search strategy in Medline: "risk factors AND (ICUAW OR critical illness neuromyopathy OR critical illness myopathy OR critical illness polyneuropathy) [limits: systematic reviews, humans, adults and English]". Titles and abstracts of the reviews obtained through the search were screened for relevance. Only relevant reviews using a systematic search strategy were included, narrative reviews were excluded. Because the most recent systematic review evaluated literature until 2006[1], we performed an additional search for original studies on risk factors for ICU-AW published in 2006 or later using the following strategy in Medline: "risk factors AND (ICUAW OR critical illness neuromyopathy OR critical illness myopathy OR critical illness polyneuropathy) [limits: published > 01-01-2006, adults, humans and English]".

Extracted relevant risk factors

For risk factor extracting only the systematic review by Stevens et al[1] was used since it was an update from the other identified systemic review[2]. Additionally risk factors were extracted from the following studies published after the most recent systematic review: Nanas et al[3], Weber-Carstens et al[4] and Anastasopoulus et al[5]. We identified the following relevant risk factors in our literature search: glucose[1,3], renal replacement therapy[1], systemic inflammatory response syndrome (SIRS)[1], sepsis[1,5], multiple organ dysfunction syndrome (MODS)[1,3,4], severity of illness[1,3,5], age[1], gender[1,5], hypocalcaemia[5],

catecholamines[1,3,4], morphine[1], corticosteroids[1], aminoglycosides[1,3], neuromuscular blockers[1], midazolam[4] and fentanyl[4].

We excluded the following risk factors because they are not easily available: insulin-like growth factor-binding protein-1[4], interleukin-6[4] and hypoalbuminemia[3]. Additionally, we excluded duration of mechanical ventilation[1], hospital length of stay[1], ICU length of stay[1] and presence of gram-negative bacteria[3] as relevant risk factors because these are not available within two days of ICU admission.

Literature for supporting material S1

1. Stevens RD, Dowdy DW, Michaels RK, Mendez-Tellez PA, Pronovost PJ, et al. (2007) Neuromuscular dysfunction acquired in critical illness: a systematic review. Intensive Care Med 33: 1876–1891.

2. De Jonghe B, Cook D, Sharshar T, Lefaucheur J-P, Carlet J, et al. (1998) Acquired neuromuscular disorders in critically ill patients: a systematic review. Groupe de Reflexion et d’Etude sur les Neuromyopathies En Reanimation. Intensive Care Med 24: 1242–1250.

3. Nanas S, Kritikos K, Angelopoulos E, Siafaka A, Tsikriki S, et al. (2008) Predisposing factors for critical illness polyneuromyopathy in a multidisciplinary intensive care unit. Acta Neurol Scand 118: 175–181.

4. Weber-Carstens S, Deja M, Koch S, Spranger J, Bubser F, et al. (2010) Risk factors in critical illness myopathy during the early course of critical illness: a prospective observational study. Crit Care 14: R119.

5. Anastasopoulos D, Kefaliakos A, Michalopoulos A (2011) Is plasma calcium concentration implicated in the development of critical illness polyneuropathy and myopathy? Crit Care 15: R247.
